# Supplementary material for: Selective molecular and network architecture features underlie brain cortical atrophy in dementia with Lewy bodies
Source: J Biomed Sci. 2026 Jun 10;33:61. doi: 10.1186/s12929-026-01267-6 (PMC13251274; doi:10.1186/s12929-026-01267-6)
Supplement: Supplementary file 1 — Supplementary material 1. [file 12929_2026_1267_MOESM1_ESM.pdf]

## Supplementary Figures

**Supplementary Figure 1**. Transcriptomic architecture of cortical thinning in DLB.

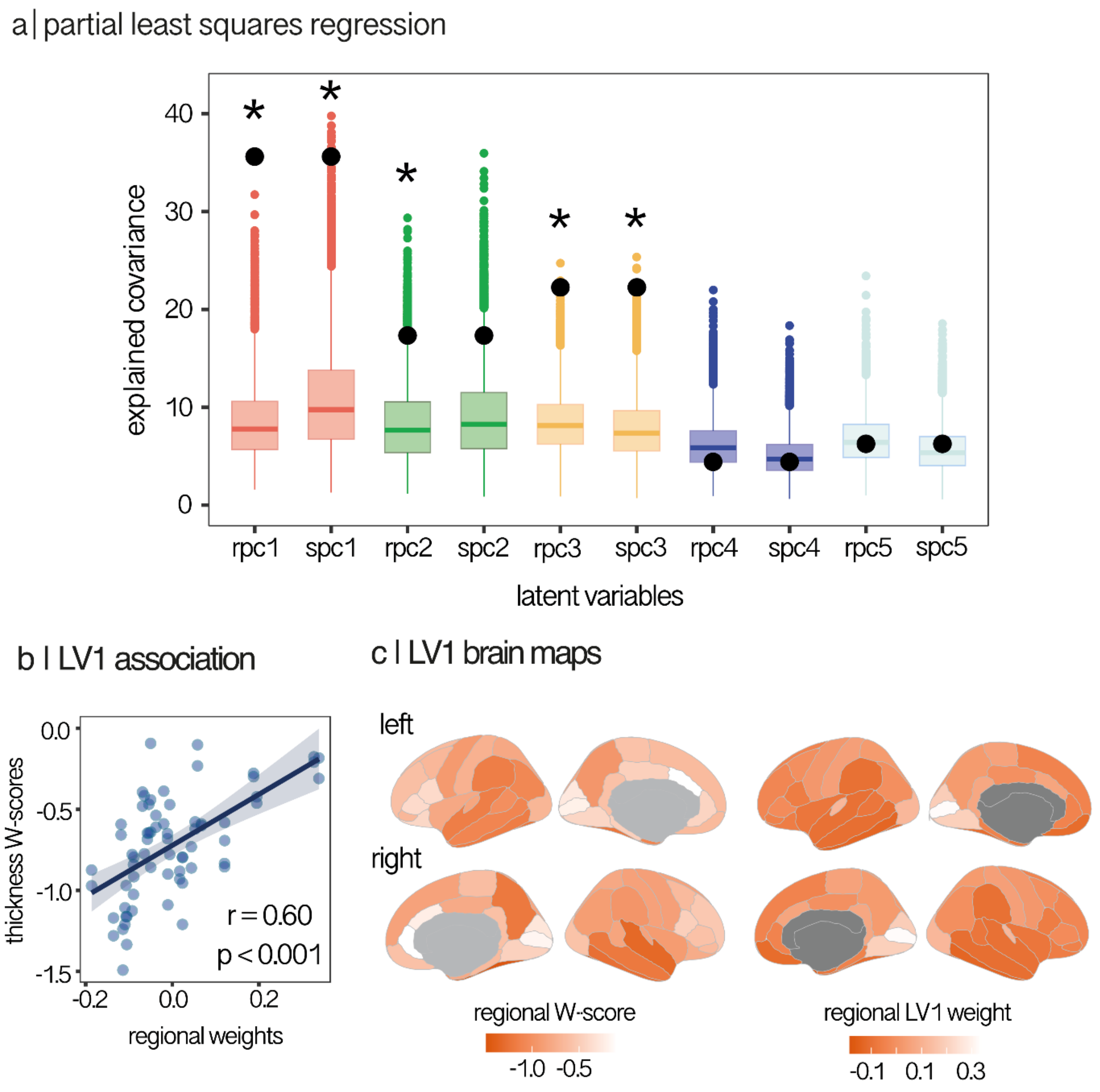


**(a)** Covariance in regional cortical thickness W-scores explained by each partial least squares LV. Boxplots show the distribution of covariance explained under 10,000 null models (random [rpc] and spatial constrained [spc]); black dots indicate empirical variance explained by observed data. Asterisks denote LVs that significantly exceeded both null distributions. **(b)** Scatterplot showing the correlation between regional cortical thickness W-scores and the gene expression weights from the first LV. **(c)** Brain surface maps showing regional distribution of cortical thickness W-scores (left) and corresponding expression weights from the first LV (right).

DLB, dementia with Lewy bodies; LV, latent variable; rpc, random null models; spc, spatial null models.

**Supplementary Figure 2.** Transcriptomics architecture in DLB for latent variable 3.

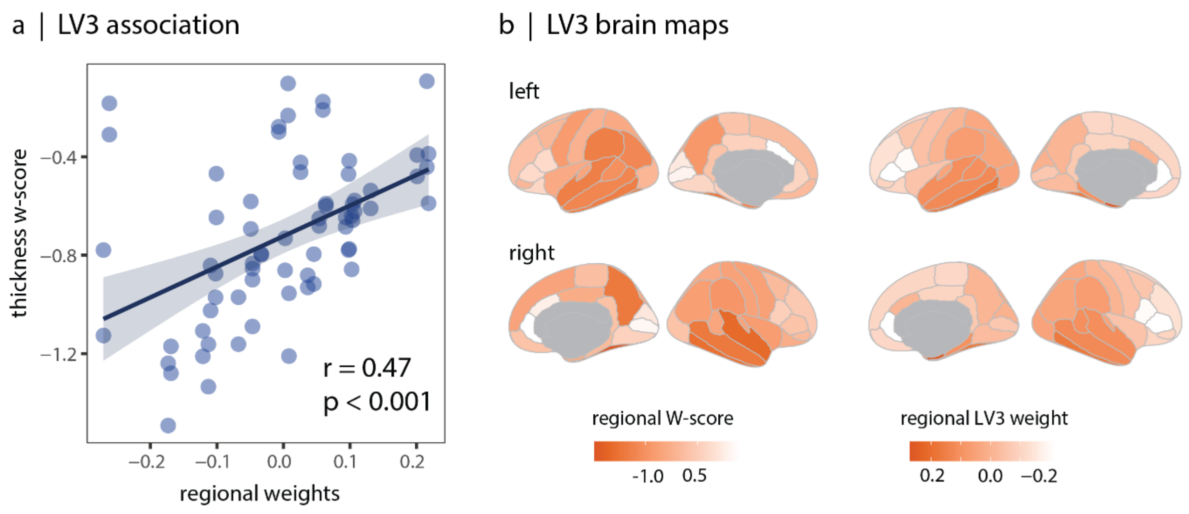


**(a)** Scatterplot showing the correlation between regional cortical thickness W-scores and the gene expression weights from the third LV. **(b)** Brain surface maps showing regional distribution of cortical thickness W-scores (left) and corresponding expression weights from the third LV (right).

**Supplementary Figure 3.** Functional enrichment of genes associated with cortical volume in DLB.

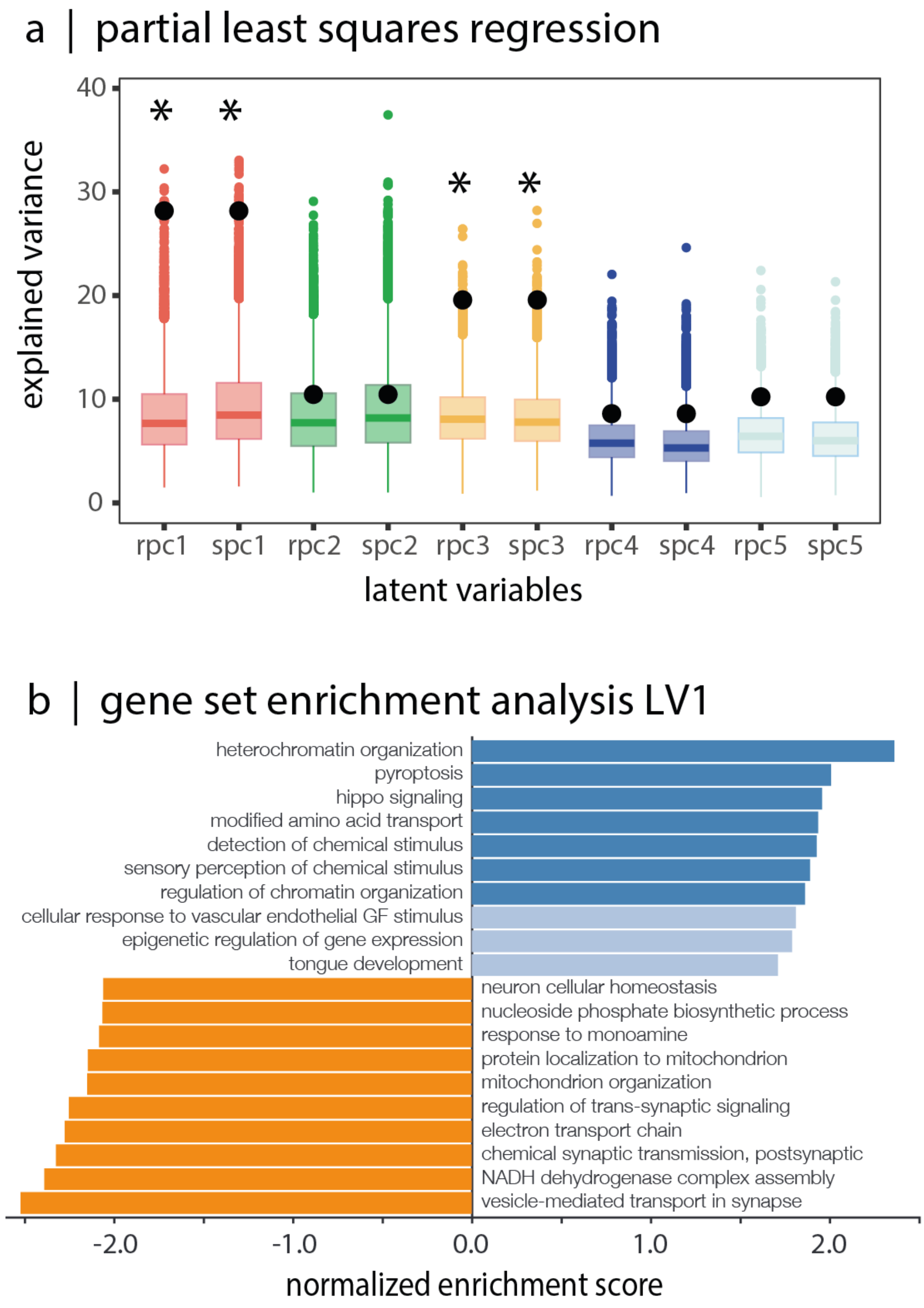


**(a)** Covariance in regional cortical volume W-scores explained by each partial least squares LV. Boxplots show the distribution of covariance explained under 10,000 null models (random [rpc] and spatial constrained [spc]); black dots indicate empirical variance explained by observed data. Asterisks denote LVs that significantly exceeded both null distributions. **(b)** GO biological process enrichment analysis of LV1. Bar plot showing the top 10 GO terms enriched

among negatively weighted genes (more highly expressed in regions with lower cortical volume; orange) and positively weighted genes (regions with relatively preserved volume; blue). Bars indicate normalized enrichment score; lighter shading denotes terms not surviving FDR correction.

DLB, dementia with Lewy bodies; FDR, false discovery rate; GO, Gene Ontology; LV, latent variable; NADH, nicotinamide adenine dinucleotide, reduced form; rpc, random null models; spc, spatial null models.
