# Supplementary material for: Selective molecular and network architecture features underlie brain cortical atrophy in dementia with Lewy bodies
Source: J Biomed Sci. 2026 Jun 10;33:61. doi: 10.1186/s12929-026-01267-6 (PMC13251274; doi:10.1186/s12929-026-01267-6)
Supplement: Supplementary file 2 — Supplementary material 2. [file 12929_2026_1267_MOESM2_ESM.pdf]

Supplementary Tables

Supplementary Table 1. Source of clinical variables for each cohort

**Clinical Variable**	**CCNA/COMPASS-ND**	**Newcastle – AMPLE**	**Newcastle – CATFIELD**	**Newcastle – TMSS**
**Literature references**	[1-4]	[5]	[6]	[7]
**Global cognitive function**	MoCA[8]	MMSE[9]	MMSE[9]	MMSE[9]
**Cognitive fluctuations**	Mayo Fluctuation Scale[10]	Dementia Cognitive Fluctuation Scale[11]	Dementia Cognitive Fluctuation Scale[11]	Dementia Cognitive Fluctuation Scale[11]
**Visual hallucinations**	NPI-Q[12]	Lewy Body Symptom Questionnaire[13]	Lewy Body Symptom Questionnaire[13]	NEVHI[14]
**REM sleep behavior disorder**	Screening question: Moves in their sleep or acts out their dreams (yes/no)	Lewy Body Symptom Questionnaire[13]	Lewy Body Symptom Questionnaire[13]	Screening question: evidence of REM behavior disorder? (yes/no)
**Parkinsonism**	Spontaneous features of parkinsonism	Lewy Body Symptom Questionnaire[13]	Lewy Body Symptom Questionnaire[13]	UPDRS, part III[15, 16]

CCNA, Canadian Consortium for Neurodegeneration in Aging; COMPASS-ND, Comprehensive Assessment of Neurodegeneration and Dementia; MMSE, Mini-Mental State Examination; MoCA, Montreal Cognitive Assessment; NEVHI, North-East Visual Hallucinations Interview; NPI-Q, Neuropsychiatric Inventory Questionnaire; UPDRS, Unified Parkinson's Disease Rating Scale

Supplementary Table 2. GTEx list of tissues analyzed.

Tissue category (brain versus body)
Brain tissue (N=13):<br>- Amygdala<br>- Anterior cingulate cortex (BA24)<br>- Caudate<br>- Cerebellar hemisphere<br>- Cerebellum<br>- Cortex<br>- Frontal cortex (BA9)<br>- Hippocampus<br>- Hypothalamus<br>- Nucleus accumbens<br>- Putamen<br>- Spinal cord, cervical C1<br>- Substantia nigra
Peripheral body tissue (N=55):<br>- Adipose tissue (subcutaneous)<br>- Adipose tissue (visceral, omentum)<br>- Adrenal gland<br>- Artery (aorta)<br>- Artery (coronary)<br>- Artery (tibial)<br>- Bladder<br>- Cervix (ectocervix)<br>- Cervix (endocervix)<br>- Colon (sigmoid)<br>- Colon (transverse)<br>- Colon (transverse, mixed cell)<br>- Colon (transverse, mucosa)<br>- Colon (transverse, muscularis)<br>- Cultured fibroblasts<br>- EBV-transformed lymphocytes<br>- Esophagus (gastroesophageal junction)<br>- Esophagus (mucosa)<br>- Esophagus (muscularis)<br>- Fallopian tube<br>- Heart (atrial appendage)<br>- Heart (left ventricle)<br>- Kidney (cortex)<br>- Kidney (medulla)<br>- Liver<br>- Liver (hepatocytes)<br>- Liver (mixed cells)<br>- Liver (portal tract)<br>- Lung<br>- Mammary gland (breast tissue)<br>- Minor salivary gland<br>- Muscle (skeletal)<br>- Nerve (tibial)<br>- Ovary<br>- Pancreas<br>- Pancreas (acini)<br>- Pancreas (islets)<br>- Pancreas (mixed cell)<br>- Pituitary gland<br>- Prostate<br>- Skin (not sun-exposed, suprapubic)

- Skin (sun-exposed, lower leg)<br>- Small intestine (terminal ileum)<br>- Small intestine (terminal ileum, lymphoid aggregate)<br>- Small intestine (terminal ileum, mixed cell)<br>- Spleen<br>- Stomach<br>- Stomach (mixed cell)<br>- Stomach (mucosa)<br>- Stomach (muscularis)<br>- Testis<br>- Thyroid gland<br>- Uterus<br>- Vagina<br>- Whole blood

BA, Brodmann area; EBV, Epstein-Barr virus; GTEx, Genotype-Tissue Expression.

Supplementary Table 3. List of the imaging studies used to generate neuromaps maps

Neurotransmitter system	Imaging modality	Study
**GABA**: GABA$_A$	[^{11}C]flumazenil PET	Norgaard et al, 2021 [17, 18]<br>Hansen et al, 2022 [18]
**Glutamate**: mGluR5	[^{11}C]ABP688 PET	DuBois et al, 2016 [19]<br>Smart et al, 2019 [20]<br>Hansen et al, 2022 [18]
**Acetylcholine**<br>• α4β2<br>• M1<br>• vesicular acetylcholine transporter [VAChT]	• [^{18}F]flubatine PET<br>• [^{11}C]LSN3172176 PET<br>• [^{18}F]FEOBV PET	Hillmer et al, 2016 [21]<br>Naganawa et al, 2021 [22]<br>Aghourian et al, 2017 [23]<br>Bedard et al, 2019 [24]<br>Hansen et al, 2022 [18]
**Dopamine**<br>• D1 receptor<br>• D2 receptor<br>• dopamine transporter [DAT]	• [^{11}C]SCH23390 PET<br>• [^{18}F]fallypride PET<br>• [^{11}C]FLB457 PET<br>• [^{11}C]raclopride PET<br>• [^{18}F]FE-PE2I PET<br>• [123I]FP-CIT SPECT	Kaller et al, 2017 [25]<br>Jaworska et al, 2020 [26]<br>Smith et al, 2019 [27]<br>Sandiego et al, 2015 [28]<br>Alakurtti et al, 2015 [29]<br>Sasaki et al, 2012 [30]<br>Marek et al, 2018 [31]<br>Hansen et al, 2022 [18]
**Noradrenaline**: noradrenaline transporter [NET]	[^{11}C]MRB PET	Hesse et al, 2017 [32]<br>Ding et al, 2010 [33]<br>Hansen et al, 2022 [18]
**Serotonin**<br>• 5-HT$_{1A}$<br>• 5-HT$_{1B}$<br>• 5-HT$_{2A}$<br>• 5-HT$_4$<br>• 5-HT$_6$<br>• serotonin transporter [5-HTT]	• [^{11}C]CUMI-101 PET<br>• [^{11}C]WAY-100635 PET<br>• [^{11}C]AZ10419369<br>• [^{11}C]P943<br>• [^{18}F]ALTANSERIN<br>• [^{11}C]CIMBI-36<br>• [^{11}C]MDL100907<br>• [^{11}C]SB207145<br>• [^{11}C]GSK215083<br>• [^{11}C]DASB	Beliveau et al, 2017 [34]<br>Savli et al, 2012 [35]<br>Beliveau et al, 2017 [34]<br>Gallezot et al, 2010 [36]<br>Savli et al, 2012 [35]<br>Beliveau et al, 2017 [34]<br>Talbot et al, 2012 [37]<br>Beliveau et al, 2017 [34]<br>Radhakrishnan et al, 2018 [38]<br>Beliveau et al, 2017 [34]<br>Savli et al, 2012 [35]<br>Hansen et al, 2022 [18]
**Endocannabinoids**: CB1	• [^{18}F]FMPEP-D2 PET<br>• [^{11}C]OMAR PET	Laurikainen et al, 2019 [39]<br>Normandin et al, 2015 [40]<br>Hansen et al, 2022 [18]
**Opioids**: μ		Hansen et al, 2022 [18]
**Histamine**: H$_3$	[^{11}C]GSK189254 PET	Gallezot et al, 2017 [41]<br>Hansen et al, 2022 [18]
**Synaptic density**: SV2A	[^{11}C]UCB-J PET	Mecca et al, 2020 [42]

		Holmes et al, 2019 [43]<br>Finnema et al, 2019 [44]<br>Chen et al, 2018 [45]<br>Hansen et al, 2022 [18]

Supplementary Table 4. Clinical characteristics of DLB patients and controls across cohorts

Clinical Assessment	Controls	DLB	P-value
**Newcastle Dataset (N = 69 DLB)**			
TMSS cohort (N = 16 DLB)			
Non-visual hallucinations, n (%)	0 (0%)	6 (43%)	**< 0.0001[a]**
Delusions, n (%)	0 (0%)	8 (53%)	**< 0.0001[a]**
Fluctuations:	0 (0%)	16 (100%)	**< 0.0001[a]**
- Impaired alertness n (%)	-	15 (94%)	NA
- Confusion, n (%)	-	13 (87%)	NA
- Total score	-	8.9 ± 4.2	NA
CATFIELD cohort (N = 21 DLB)			
Fluctuations:	-	13 (68%)	NA
- Impaired alertness, n (%)	-	13 (68%)	NA
- Confusion, n (%)	-	12 (63%)	NA
- Total score	-	4.1 ± 4.2	NA
AMPLE cohort (N = 32 DLB)			
Parkinsonian motor features:			
- Rigidity, n (%)	2 (12%)	12 (39%)	**< 0.0001[a]**
- Shuffling gait, n (%)	0 (0%)	26 (84%)	**< 0.0001[a]**
- Tremor, n (%)	3 (18%)	25 (81%)	**< 0.0001[a]**
- Slowness, n (%)	0 (0%)	24 (80%)	**< 0.0001[a]**
- Hypomimia, n (%)	0 (0%)	16 (53%)	**< 0.0001[a]**
- Balance problems, n (%)	2 (15%)	22 (73%)	**< 0.0001[a]**
Loss of smell, n (%)	3 (18%)	18 (58%)	**< 0.0001[a]**
Fluctuations:	-	26 (81%)	NA
- Impaired alertness, n (%)	-	22 (69%)	NA
- Confusion, n (%)	-	20 (63%)	NA
- Total score	-	6.6 ± 4.3	NA
Auditory hallucinations, n (%)	0 (0%)	8 (28%)	**< 0.0001[a]**
Sleep abnormalities:			
- Dream enactment behaviors, n (%)	1 (6%)	16 (55%)	**< 0.0001[a]**

- Restless legs syndrome, n (%)	1 (6%)	5 (16%)	**0.04[a]**
**CCNA/COMPASS-ND Dataset (N = 14 DLB)**			
PDQ-39 score	-	27.5 ± 15.5	NA
MBI-C score	0.1 ± 0.4	18.5 ± 9.7	**< 0.0001[b]**
NPI-Q score:			
- Severity score	0.7 ± 1.2	8.9 ± 6.8	**< 0.0001[b]**
- Distress score	0.5 ± 1.2	10.0 ± 8.9	**< 0.0001[b]**
GAD-7 anxiety scale	3.2 ± 4.0	6.8 ± 5.0	**0.01**
GDS score			
CDR scale score, median (IQR)	0 (0)	-	NA
Cognitive testing:			
- RAVLT, trial A5, z-score	0.9 ± 0.8	-1.4 ± 1.5	**< 0.0001[b]**
- RAVLT, trial A7, z-score	1.4 ± 1.8	-1.0 ± 1.3	**< 0.0001[b]**
- Trail Making Test, part A, z-score	-0.7 ± 0.6	2.5 ± 1.7	**< 0.0001[b]**
- Trail Making Test, part B, z-score	-0.3 ± 1.1	3.1 ± 1.5	**< 0.0001[b]**
- Letter fluency, sum of words	46 ± 10	21 ± 9	**< 0.0001[b]**
- Judgement of Line Orientation, score	26 ± 4	15 ± 8	**< 0.0001[b]**

Data are presented as mean ± standard deviation unless otherwise indicated. Bold values represent significant differences. [a] Fisher's exact test; [b] Student's t-test

CCNA, Canadian Consortium for Neurodegeneration in Aging; CDR, Clinical Dementia Rating; COMPASS-ND, Comprehensive Assessment of Neurodegeneration and Dementia; DLB, dementia with Lewy bodies; GAD-7, Generalized Anxiety Disorder; GDS, Geriatric Depression Scale; IQR, interquartile range; MBI-C, Mild Behavioral Impairment Checklist; MMSE, Mini-Mental State Examination; MoCA, Montreal Cognitive Assessment; NA not available; NPI-Q, Neuropsychiatric Inventory Questionnaire; PDQ-39, 39-item Parkinson's Disease Questionnaire; RAVLT, Rey Auditory Verbal Learning Test.

Supplementary Table 5. Region-wise cortical thickness comparisons between DLB patients and controls

Region	Controls	DLB	P-value	P_{FDR}-value	*W*-score DLB
**Left hemisphere**					
banks superior temp. sulcus	2.38±0.11	2.27±0.16	<0.00001	**0.00001**	-1.026
caudal anterior cingulate	2.34±0.19	2.30±0.24	0.46859	0.47558	-0.102
caudal middle frontal	2.46±0.11	2.38±0.18	0.00234	**0.00370**	-0.681
cuneus	1.84±0.13	1.80±0.16	0.17945	0.19682	-0.278
entorhinal	3.04±0.27	2.73±0.37	<0.00001	**<0.00001**	-1.127
fusiform	2.57±0.14	2.40±0.24	<0.00001	**<0.00001**	-1.239
inferior parietal	2.34±0.09	2.24±0.12	<0.00001	**<0.00001**	-1.090
inferior temporal	2.62±0.13	2.46±0.19	<0.00001	**<0.00001**	-1.170
isthmus cingulate	2.13±0.13	2.05±0.17	0.00270	**0.00418**	-0.590
lateral occipital	2.16±0.11	2.08±0.14	0.00011	**0.00026**	-0.693
lateral orbitofrontal	2.54±0.11	2.49±0.21	0.11895	0.13481	-0.416
lingual	1.88±0.12	1.82±0.16	0.02015	**0.02586**	-0.423
medial orbitofrontal	2.26±0.14	2.17±0.19	0.00086	**0.00151**	-0.658
middle temporal	2.66±0.12	2.53±0.17	<0.00001	**<0.00001**	-1.108
parahippocampal	2.58±0.26	2.45±0.28	0.00348	**0.00504**	-0.469
paracentral	2.24±0.17	2.14±0.25	0.00592	**0.00805**	-0.578
pars opercularis	2.47±0.10	2.42±0.15	0.08042	0.09429	-0.393
pars orbitalis	2.60±0.15	2.50±0.19	0.00026	**0.00056**	-0.685
pars triangularis	2.34±0.10	2.30±0.12	0.04465	0.05327	-0.388
pericalcarine	1.60±0.13	1.57±0.15	0.28816	0.30462	-0.210
postcentral	2.04±0.12	1.95±0.14	0.00006	**0.00016**	-0.731
posterior cingulate	2.27±0.15	2.19±0.21	0.01936	**0.02531**	-0.471
precentral	2.43±0.14	2.30±0.23	0.00006	**0.00016**	-0.931
precuneus	2.27±0.12	2.15±0.17	<0.00001	**0.00001**	-0.955
rostral anterior cingulate	2.57±0.21	2.48±0.25	0.02296	**0.02892**	-0.441
rostral middle frontal	2.34±0.10	2.27±0.12	0.00074	**0.00132**	-0.610
superior frontal	2.56±0.12	2.48±0.17	0.00181	**0.00293**	-0.641
superior parietal	2.18±0.11	2.09±0.13	0.00001	**0.00004**	-0.795
superior temporal	2.57±0.12	2.43±0.18	<0.00001	**<0.00001**	-1.163
supramarginal	2.42±0.10	2.31±0.13	<0.00001	**<0.00001**	-1.162
frontal pole	2.73±0.25	2.66±0.26	0.10146	0.11694	-0.310
temporal pole	3.41±0.28	3.15±0.45	0.00010	**0.00025**	-0.875
transverse temporal	2.30±0.22	2.10±0.27	0.00001	**0.00002**	-0.834
insula	2.73±0.15	2.61±0.24	0.00034	**0.00070**	-0.796
**Right hemisphere**					
banks superior temp. sulcus	2.46±0.13	2.34±0.16	0.00001	**0.00004**	-0.842
caudal anterior cingulate	2.26±0.19	2.21±0.20	0.18525	0.19995	-0.232
caudal middle frontal	2.46±0.12	2.37±0.15	0.00037	**0.00074**	-0.650
cuneus	1.84±0.12	1.81±0.16	0.12367	0.13786	-0.299
entorhinal	3.00±0.30	2.76±0.38	0.00005	**0.00013**	-0.779
fusiform	2.57±0.13	2.38±0.24	<0.00001	**<0.00001**	-1.492
inferior parietal	2.36±0.09	2.27±0.15	0.00003	**0.00008**	-0.900
inferior temporal	2.64±0.13	2.47±0.20	<0.00001	**<0.00001**	-1.280
isthmus cingulate	2.12±0.16	2.02±0.18	0.00053	**0.00098**	-0.600
lateral occipital	2.21±0.12	2.14±0.14	0.00108	**0.00184**	-0.581
lateral orbitofrontal	2.47±0.11	2.38±0.19	0.00135	**0.00224**	-0.775
lingual	1.90±0.11	1.84±0.17	0.02641	**0.03207**	-0.462
medial orbitofrontal	2.31±0.14	2.23±0.21	0.00708	**0.00944**	-0.592
middle temporal	2.70±0.10	2.57±0.18	<0.00001	**<0.00001**	-1.211
parahippocampal	2.52±0.22	2.36±0.27	0.00038	**0.00074**	-0.647
paracentral	2.23±0.16	2.13±0.24	0.00454	**0.00643**	-0.622

pars opercularis	2.46±0.13	2.39±0.19	0.02372	**0.02933**	-0.480
pars orbitalis	2.56±0.17	2.45±0.24	0.00339	**0.00501**	-0.647
pars triangularis	2.35±0.13	2.27±0.19	0.00556	**0.00772**	-0.589
pericalcarine	1.62±0.13	1.59±0.17	0.45280	0.46652	-0.176
postcentral	2.04±0.12	1.93±0.15	0.00001	**0.00002**	-0.862
posterior cingulate	2.28±0.13	2.18±0.20	0.00045	**0.00085**	-0.779
precentral	2.40±0.14	2.28±0.24	0.00021	**0.00045**	-0.882
precuneus	2.28±0.11	2.14±0.16	<0.00001	**<0.00001**	-1.210
rostral anterior cingulate	2.65±0.20	2.64±0.27	0.79213	0.79213	-0.093
rostral middle frontal	2.32±0.13	2.25±0.13	0.00304	**0.00459**	-0.538
superior frontal	2.56±0.11	2.47±0.16	0.00012	**0.00028**	-0.858
superior parietal	2.18±0.11	2.08±0.14	0.00002	**0.00007**	-0.798
superior temporal	2.62±0.13	2.45±0.20	<0.00001	**<0.00001**	-1.335
supramarginal	2.43±0.11	2.32±0.14	<0.00001	**<0.00001**	-0.970
frontal pole	2.68±0.24	2.63±0.27	0.29118	0.30462	-0.183
temporal pole	3.41±0.28	3.12±0.51	0.00007	**0.00016**	-0.972
transverse temporal	2.32±0.21	2.13±0.26	0.00001	**0.00002**	-0.855
insula	2.74±0.16	2.58±0.26	0.00003	**0.00008**	-0.917

Mean ± standard deviation cortical thickness values are shown for the DLB and control groups across each cortical region following data harmonization using ComBAT. Group differences were assessed using analysis of variance (ANOVA) adjusted for age and sex. FDR-corrected P-values (P_{FDR}) are reported, with statistical significance set at $P_{FDR} < 0.05$. *W*-scores represent standardized deviations in cortical thickness for DLB patients relative to age- and sex-matched controls as reference; negative values indicate regional cortical thinning. Regions are listed by hemisphere and anatomical label according to the Desikan-Killiany atlas.

DLB, dementia with Lewy bodies; FDR, false discovery rate.

Supplementary Table 6. Region-wise cortical volume comparisons between DLB patients and controls

Region	Controls	DLB	P-value	P_{FDR}-value	*W*-score DLB
**Left hemisphere**					
banks superior temp. sulcus	2198.39 ± 367.88	2045.42 ± 373.57	0.00035	**0.00059**	-0.487
caudal anterior cingulate	1561.96 ± 379.13	1544.24 ± 490.17	0.32185	0.33670	-0.053
caudal middle frontal	5752.86 ± 908.43	5447.13 ± 1036.44	0.00198	**0.00274**	-0.351
cuneus	2777.84 ± 2767.37	2767.37 ± 561.42	0.38215	0.39373	-0.066
entorhinal	1802.56 ± 350.46	1560.44 ± 374.36	<0.00001	**<0.00001**	-0.755
fusiform	8756.32 ± 1111.07	7982.31 ± 1419.31	<0.00001	**<0.00001**	-0.891
inferior parietal	10724.97 ± 1567.56	9689.82 ± 1574.99	<0.00001	**<0.00001**	-0.756
inferior temporal	10039.56 ± 1305.62	9161.56 ± 1472.28	<0.00001	**<0.00001**	-0.856
isthmus cingulate	2405.87 ± 335.37	2300.95 ± 430.40	0.00072	**0.00114**	-0.444
lateral occipital	11446.38 ± 1472.07	10713.24 ± 1495.44	<0.00001	**<0.00001**	-0.562
lateral orbitofrontal	7104.23 ± 852.21	6880.17 ± 1012.89	0.00002	**0.00004**	-0.408
lingual	5759.99 ± 958.69	5493.39 ± 1004.88	0.00701	**0.00860**	-0.293
medial orbitofrontal	4707.62 ± 618.34	4578.81 ± 746.12	0.00403	**0.00527**	-0.315
middle temporal	9702.73 ± 1314.31	8972.94 ± 1325.81	<0.00001	**<0.00001**	-0.690
parahippocampal	1940.88 ± 344.57	1799.90 ± 359.86	0.00045	**0.00072**	-0.429
paracentral	3196.96 ± 382.78	3036.32 ± 512.86	0.00115	**0.00177**	-0.412
pars opercularis	4292.37 ± 760.29	4063.72 ± 702.29	0.00287	**0.00382**	-0.408
pars orbitalis	2201.96 ± 290.84	2043.49 ± 327.57	<0.00001	**<0.00001**	-0.705
pars triangularis	3254.94 ± 474.46	3140.26 ± 539.74	0.00564	**0.00710**	-0.380
pericalcarine	1870.63 ± 326.89	1910.62 ± 451.67	0.82524	0.83756	0.107
postcentral	9196.90 ± 1115.75	8717.50 ± 1294.13	0.00002	**0.00004**	-0.497
posterior cingulate	2796.80 ± 418.83	2541.51 ± 576.20	0.00001	**0.00002**	-0.729
precentral	12550.04 ± 1524.54	11705.86 ± 1785.43	<0.00001	**<0.00001**	-0.618
precuneus	8792.49 ± 1101.44	8117.16 ± 1234.69	<0.00001	**<0.00001**	-0.773
rostral anterior cingulate	2432.15 ± 507.87	2371.58 ± 564.84	0.02505	**0.02989**	-0.176
rostral middle frontal	13559.13 ± 1722.91	13526.60 ± 2073.53	0.07051	0.07991	-0.041
superior frontal	20614.95 ± 2324.36	20167.58 ± 2768.83	0.00120	**0.00181**	-0.270
superior parietal	12267.58 ± 1349.25	11401.96 ± 1651.01	<0.00001	**<0.00001**	-0.790
superior temporal	11356.18 ± 1281.80	10713.88 ± 1586.83	<0.00001	**<0.00001**	-0.605
supramarginal	10293.77 ± 1554.89	9883.23 ± 1647.49	0.00025	**0.00046**	-0.374
frontal pole	1004.79 ± 162.76	946.31 ± 172.46	0.00708	**0.00860**	-0.460
temporal pole	2544.93 ± 350.89	2354.55 ± 495.54	0.00147	**0.00212**	-0.550
transverse temporal	1183.14 ± 228.00	1051.67 ± 250.60	<0.00001	**0.00001**	-0.591
insula	6781.53 ± 821.50	5369.40 ± 998.65	<0.00001	**<0.00001**	-0.660
**Right hemisphere**					
banks superior temp. sulcus	1995.05 ± 159.50	1783.17 ± 287.39	<0.00001	**<0.00001**	-0.849
caudal anterior cingulate	1813.58 ± 436.32	1741.89 ± 436.45	0.09864	0.10819	-0.168
caudal middle frontal	5414.61 ± 817.71	5333.69 ± 1118.88	0.04969	0.05727	-0.092
cuneus	3008.05 ± 406.70	3982.31 ± 580.68	0.25711	0.27318	-0.083
entorhinal	1647.16 ± 393.60	1469.64 ± 365.99	0.00133	**0.00197**	-0.414
fusiform	8471.71 ± 1168.03	7518.18 ± 1425.77	<0.00001	**<0.00001**	-0.971
inferior parietal	13102.84 ± 1967.61	11768.20 ± 1909.55	<0.00001	**<0.00001**	-0.802
inferior temporal	9591.39 ± 1254.19	8794.38 ± 1563.81	<0.00001	**<0.00001**	-0.732
isthmus cingulate	2164.67 ± 349.76	2060.17 ± 429.04	0.00555	**0.00710**	-0.370
lateral occipital	11667.40 ± 1495.83	11176.67 ± 1868.71	0.00015	**0.00028**	-0.385
lateral orbitofrontal	6872.25 ± 798.16	6642.13 ± 1009.91	0.00001	**0.00002**	-0.424
lingual	6069.13 ± 937.40	5923.23 ± 1076.43	0.08567	0.09550	-0.170
medial orbitofrontal	5230.08 ± 664.45	5021.89 ± 840.39	0.00006	**0.00012**	-0.440
middle temporal	10668.87 ± 1151.51	9888.43 ± 1570.56	<0.00001	**<0.00001**	-0.919
parahippocampal	1777.14 ± 252.29	1661.88 ± 322.37	0.00014	**0.00028**	-0.507

paracentral	3509.08 ± 492.70	3334.08 ± 598.98	0.00149	**0.00212**	-0.411
pars opercularis	3513.36 ± 571.92	3298.93 ± 545.99	0.00009	**0.00018**	-0.486
pars orbitalis	2516.43 ± 309.74	2386.70 ± 418.47	0.00031	**0.00055**	-0.556
pars triangularis	3854.28 ± 663.92	3580.09 ± 619.03	0.00003	**0.00006**	-0.546
pericalcarine	2138.98 ± 361.14	2194.08 ± 528.18	0.89068	0.89068	0.095
postcentral	8819.50 ± 1187.47	8316.13 ± 1378.42	0.00001	**0.00002**	-0.484
posterior cingulate	2809.69 ± 412.55	2633.60 ± 633.96	0.00264	**0.00359**	-0.479
precentral	12409.06 ± 1398.83	11615.66 ± 1931.38	<0.00001	**<0.00001**	-0.644
precuneus	9201.89 ± 1139.97	8447.43 ± 1335.48	<0.00001	**<0.00001**	-0.825
rostral anterior cingulate	1820.10 ± 349.45	1738.27 ± 433.41	0.03128	**0.03668**	-0.296
rostral middle frontal	14178.55 ± 2003.91	13724.47 ± 2212.94	0.00036	**0.00059**	-0.327
superior frontal	19796.37 ± 2279.32	19252.58 ± 2653.23	0.00024	**0.00045**	-0.324
superior parietal	12105.47 ± 1246.29	11234.78 ± 1581.68	<0.00001	**<0.00001**	-0.833
superior temporal	10796.56 ± 1202.26	10023.39 ± 1487.86	<0.00001	**<0.00001**	-0.744
supramarginal	9229.47 ± 1288.52	8731.52 ± 1441.15	<0.00001	**0.00001**	-0.529
frontal pole	1199.55 ± 197.45	1160.29 ± 237.62	0.10306	0.11124	-0.240
temporal pole	2446.62 ± 374.74	2158.83 ± 474.74	<0.00001	**0.00001**	-0.777
transverse temporal	888.61 ± 163.52	789.11 ± 159.10	<0.00001	**<0.00001**	-0.645
insula	6587.56 ± 795.24	6121.98 ± 962.01	<0.00001	**<0.00001**	-0.776

Mean ± standard deviation cortical volume values are shown for the DLB and control groups across each cortical region following data harmonization using ComBAT. Group differences were assessed using analysis of variance (ANOVA) adjusted for age, sex, and eTIV. FDR-corrected P-values (P_{FDR}) are reported, with statistical significance set at $P_{FDR} < 0.05$. W-scores represent standardized deviations in cortical volume for DLB patients relative to age- and sex-matched controls as reference; negative values indicate reduced cortical volume. Regions are listed by hemisphere and anatomical label according to the Desikan-Killiany atlas.

DLB, dementia with Lewy bodies; eTIV, estimated total intracranial volume; FDR = false discovery rate.

Supplementary Table 7. Region-wise cortical surface area comparisons between DLB patients and controls

Region	Controls	DLB	P-value	P_{FDR}-value	*W*-score DLB
**Left hemisphere**					
banks superior temp. sulcus	957.28±136.80	945.28±149.81	0.19168	0.33421	-0.152
caudal anterior cingulate	621.44±131.32	631.47±180.81	0.57657	0.75398	0.034
caudal middle frontal	2124.51±309.20	2115.50±385.00	0.33717	0.48831	0.003
cuneus	1416.19±206.07	1432.40±236.89	0.75496	0.86795	0.030
entorhinal	431.26±90.66	439.99±96.41	0.76584	0.86795	0.074
fusiform	3003.81±379.10	2960.95±438.95	0.00646	**0.03447**	-0.227
inferior parietal	4195.83±581.72	3994.85±564.14	0.00016	**0.00363**	-0.438
inferior temporal	3255.48±476.54	3234.17±511.54	0.07939	0.16359	-0.145
isthmus cingulate	1023.31±144.92	1038.80±185.23	0.38733	0.53752	-0.016
lateral occipital	4750.93±603.78	4660.02±655.09	0.03224	0.10440	-0.181
lateral orbitofrontal	2616.58±280.83	2600.72±361.52	0.04719	0.12394	-0.106
lingual	2877.57±390.60	2832.44±378.53	0.10936	0.21872	-0.132
medial orbitofrontal	1908.49±191.39	1952.80±251.54	0.81028	0.87459	0.187
middle temporal	3043.59±379.80	2985.88±412.03	0.01270	0.05134	-0.258
parahippocampal	642.82±86.17	631.42±87.75	0.01640	0.05869	-0.170
paracentral	1329.61±150.81	1368.16±177.14	0.74376	0.86795	0.238
pars opercularis	1564.44±263.59	1521.32±262.49	0.04403	0.12394	-0.273
pars orbitalis	685.99±90.84	669.42±98.98	0.00659	**0.03447**	-0.258
pars triangularis	1268.52±192.70	1250.41±195.37	0.04415	0.12394	-0.240
pericalcarine	1292.58±224.62	1325.76±243.14	0.60812	0.78023	0.145
postcentral	4067.11±415.68	4095.70±521.07	0.17309	0.30974	-0.010
posterior cingulate	1137.50±164.07	1095.12±220.87	0.01347	0.05134	-0.354
precentral	4817.64±523.66	4865.52±523.78	0.32784	0.48831	0.051
precuneus	3671.07±458.34	3599.96±444.95	0.00055	**0.00622**	-0.281
rostral anterior cingulate	842.57±178.21	867.26±201.28	0.54186	0.72248	0.077
rostral middle frontal	5098.91±662.22	5260.56±839.09	0.79739	0.87456	0.301
superior frontal	7027.73±785.72	7155.17±876.77	0.48992	0.66629	0.150
superior parietal	5119.60±547.97	4982.86±639.43	0.00031	**0.00527**	-0.365
superior temporal	3909.90±433.72	3944.19±458.42	0.26367	0.41697	0.037
supramarginal	3906.26±590.96	3970.62±601.63	0.33751	0.48831	0.037
frontal pole	271.27±35.18	258.12±28.92	0.00064	**0.00622**	-0.456
temporal pole	497.07±60.39	505.49±79.89	0.93162	0.94552	0.086
transverse temporal	456.11±63.35	454.79±76.09	0.20167	0.34284	-0.047
insula	2450.83±274.20	2428.01±253.46	0.00451	**0.03067**	-0.172
**Right hemisphere**					
banks superior temp. sulcus	856.19±99.57	801.94±115.53	0.00011	**0.00363**	-0.600
caudal anterior cingulate	712.17±158.49	710.74±165.95	0.37015	0.52438	-0.041
caudal middle frontal	1989.90±260.96	2065.08±404.10	0.67494	0.84272	0.311
cuneus	1513.91±196.07	1538.70±257.98	0.97694	0.97694	0.116
entorhinal	394.87±81.95	403.41±86.47	0.89538	0.93671	0.142
fusiform	2903.60±382.39	2842.48±404.58	0.00225	**0.01700**	-0.232
inferior parietal	5023.82±747.21	4692.54±667.08	<0.00001	**0.00007**	-0.544
inferior temporal	3094.49±426.58	3066.63±522.51	0.05211	0.13124	-0.138
isthmus cingulate	913.72±142.13	930.09±175.55	0.70640	0.84272	0.047
lateral occipital	4722.49±589.47	4678.01±758.22	0.07335	0.16199	-0.107
lateral orbitofrontal	2640.75±314.26	2676.76±358.97	0.25216	0.40826	0.075
lingual	2998.92±404.06	3025.76±415.41	0.79400	0.87456	0.047
medial orbitofrontal	2051.63±248.65	2071.26±253.88	0.16595	0.30499	0.037
middle temporal	3316.82±355.46	3268.08±474.87	0.00936	**0.04546**	-0.245
parahippocampal	610.10±82.10	608.60±77.93	0.03677	0.11365	-0.135

paracentral	1493.06±188.35	1531.16±200.96	0.68642	0.84272	0.118
pars opercularis	1315.68±201.39	1281.29±196.31	0.01079	**0.04891**	-0.288
pars orbitalis	812.64±101.59	819.46±127.02	0.27646	0.42726	-0.002
pars triangularis	1482.77±238.63	1440.02±213.39	0.00529	**0.03270**	-0.300
pericalcarine	1440.65±218.39	1475.67±280.59	0.84038	0.89290	0.111
postcentral	3926.35±458.50	3955.00±512.20	0.06952	0.16199	-0.023
posterior cingulate	1125.94±168.13	1112.19±240.91	0.21566	0.35768	-0.097
precentral	4842.46±490.95	4864.68±556.25	0.12956	0.25172	-0.004
precuneus	3845.45±483.28	3769.53±507.95	0.00049	**0.00622**	-0.276
rostral anterior cingulate	601.60±109.86	583.93±138.27	0.07504	0.16199	-0.180
rostral middle frontal	5401.56±817.91	5415.89±815.74	0.07623	0.16199	-0.007
superior frontal	6716.78±752.37	6864.73±894.21	0.69582	0.84272	0.195
superior parietal	5025.60±548.57	4874.97±656.40	0.00074	**0.00629**	-0.372
superior temporal	3647.26±370.73	3672.65±469.85	0.15813	0.29869	-0.028
supramarginal	3514.06±468.35	3502.43±530.51	0.04739	0.12394	-0.128
frontal pole	332.11±40.57	323.94±43.02	0.01359	0.05134	-0.307
temporal pole	483.04±73.35	488.81±75.23	0.91225	0.93989	0.064
transverse temporal	335.40±47.10	334.64±47.50	0.07416	0.16199	-0.134
insula	2360.57±306.92	2360.33±300.16	0.02567	0.08728	-0.126

Mean ± standard deviation cortical surface area values are shown for the DLB and control groups across each cortical region following data harmonization using ComBAT. Group differences were assessed using analysis of variance (ANOVA) adjusted for age, sex, and eTIV. FDR-corrected P-values (P_{FDR}) are reported, with statistical significance set at $P_{FDR} < 0.05$. W-scores represent standardized deviations in cortical surface area for DLB patients relative to age- and sex-matched controls as reference; negative values indicate decreased surface area. Regions are listed by hemisphere and anatomical label according to the Desikan-Killiany atlas.

DLB, dementia with Lewy bodies; eTIV, estimated total intracranial volume; FDR = false discovery rate.

Supplementary Table 8. Biological processes and cellular components enriched in regions with cortical thinning in DLB within latent variable 3

GO identifier	GO term	Gene set size	Number of leading edge IDs	ES	NES	*P*-value (FDR)
**Biological processes**						
Negatively weighted genes (associated with decreased thickness)						
GO:0099003 §	vesicle-mediated transport in synapse	205	83	-0.48	-2.61	**< 0.0001**
GO:0010257 §	NADH dehydrogenase complex assembly	54	38	-0.56	-2.39	0.0005
GO:0048499	synaptic vesicle membrane organization	27	15	-0.65	-2.33	0.001
GO:0022900 §	electron transport chain	146	88	-0.46	-2.33	0.0008
GO:0099177 §	regulation of trans-synaptic signalling	425	152	-0.38	-2.26	0.0009
GO:0099565 §	chemical synaptic transmission, postsynaptic	94	42	-0.46	-2.22	0.001
GO:0099072 §	regulation of postsynaptic membrane neurotransmitter receptor levels	82	36	-0.45	-2.14	0.002
GO:1902414 §	protein localization to cell junction	103	47	-0.43	-2.13	0.002
GO:0070585 §	protein localization to mitochondrion	122	57	-0.42	-2.12	0.002
GO:0015980 §	energy derivation by oxidation of organic compounds	280	134	-0.36	-2.02	0.005
GO:0002209 §	behavioral defense response	41	24	-0.50	-2.01	0.007
GO:0050803 §	regulation of synapse structure or activity	225	93	-0.36	-1.98	0.008
GO:0035249	synaptic transmission, glutamatergic	97	36	-0.41	-1.97	0.008
GO:0098664	G protein-coupled serotonin receptor signaling pathway	19	8	-0.61	-1.97	0.008
GO:0050808 §	synapse organization	440	172	-0.33	-1.93	0.010
GO:0070050 §	neuron cellular homeostasis	55	26	-0.45	-1.92	0.010
GO:0007005 §	mitochondrion organization	439	200	-0.32	-1.87	0.015
GO:0051703 §	biological process involved in intraspecies interaction between organisms	45	17	-0.46	-1.86	0.016
GO:1901293 §	nucleoside phosphate biosynthetic process	240	102	-0.33	-1.84	0.019
GO:0051648 §	vesicle localization	203	71	-0.34	-1.83	0.019
GO:1902600 §	proton transmembrane transport	114	67	-0.37	-1.81	0.023
GO:0042391 §	regulation of membrane potential	364	133	-0.31	-1.81	0.023
GO:0072522 §	purine-containing compound biosynthetic process	216	93	-0.33	-1.80	0.023

GO:0018210	peptidyl-threonine modification	79	24	-0.38	-1.80	0.023
GO:0050890 §	cognition	277	124	-0.32	-1.79	0.022
GO:0009141 §	nucleoside triphosphate metabolic process	223	98	-0.33	-1.79	0.021
GO:0048857 §	neural nucleus development	58	26	-0.41	-1.79	0.021
GO:0006836 §	neurotransmitter transport	178	57	-0.33	-1.77	0.024
GO:0031644 §	regulation of nervous system process	96	34	-0.36	-1.76	0.027
GO:0106027	neuron projection organization	81	37	-0.37	-1.73	0.035
GO:0001963	synaptic transmission, dopaminergic	23	10	-0.51	-1.73	0.034
GO:1990089	response to nerve growth factor	44	14	-0.42	-1.72	0.035
GO:0019693 §	ribose phosphate metabolic process	396	163	-0.29	-1.72	0.035
GO:0023019	signal transduction involved in regulation of gene expression	15	6	-0.55	-1.69	0.041
GO:0090150 §	establishment of protein localization to membrane	244	83	-0.30	-1.68	0.045
GO:0009308	amine metabolic process	90	36	-0.35	-1.66	0.048
GO:0006887 §	exocytosis	289	76	-0.29	-1.66	0.048
GO:0015844	monoamine transport	70	25	-0.37	-1.66	0.047
Positively weighted genes (associated with increased thickness)						
GO:0070828	heterochromatin organization	88	40	0.47	2.12	0.009
**Cellular components**						
Negatively weighted genes (associated with decreased thickness)						
GO:0070469	respirasome	86	62	-0.54	-2.60	< 0.0001
GO:0098798 §	mitochondrial protein-containing complex	273	164	-0.43	-2.41	< 0.0001
GO:1990204 §	oxidoreductase complex	107	66	-0.49	-2.41	< 0.0001
GO:0098984 §	neuron to neuron synapse	339	123	-0.40	-2.30	< 0.0001
GO:0098978 §	glutamatergic synapse	390	159	-0.40	-2.29	< 0.0001
GO:0099572 §	postsynaptic specialization	320	111	-0.38	-2.16	0.0004
GO:0070069 §	cytochrome complex	33	24	-0.56	-2.14	0.0006
GO:0097060 §	synaptic membrane	354	122	-0.37	-2.12	0.001
GO:0005743 §	mitochondrial inner membrane	431	215	-0.35	-2.10	0.001
GO:0060076 §	excitatory synapse	62	26	-0.47	-2.06	0.001
GO:0098685 §	Schaffer collateral - CA1 synapse	90	45	-0.42	-1.99	0.002
GO:0048786 §	presynaptic active zone	71	30	-0.45	-1.98	0.002
GO:0043198 §	dendritic shaft	40	24	-0.48	-1.97	0.002
GO:0150034 §	distal axon	242	96	-0.34	-1.90	0.004
GO:0044309 §	neuron spine	165	60	-0.36	-1.86	0.006
GO:1990351 §	transporter complex	368	113	-0.31	-1.82	0.004
GO:0044306 §	neuron projection terminus	111	40	-0.37	-1.80	0.010
GO:0030133 §	transport vesicle	376	110	-0.30	-1.77	0.012
GO:0030427 §	site of polarized growth	153	59	-0.34	-1.77	0.012
GO:0071782	endoplasmic reticulum tubular network	26	13	-0.47	-1.74	0.015
GO:0005759 §	mitochondrial matrix	451	199	-0.29	-1.71	0.018
GO:0098982 §	GABA-ergic synapse	74	23	-0.37	-1.70	0.019
GO:0043025 §	neuronal cell body	433	151	-0.28	-1.66	0.026
GO:0099568 §	cytoplasmic region	223	86	-0.30	-1.66	0.025

GO:0031045	dense core granule	34	9	-0.43	-1.62	0.031
GO:0048770 §	pigment granule	103	32	-0.34	-1.62	0.031
GO:0099522 §	cytosolic region	19	14	-0.47	-1.56	0.047
GO:1905368 §	peptidase complex	111	45	-0.31	-1.56	0.047

List of genes significantly enriched in biological processes and cellular components within the third latent variable for cortical thickness based on gene enrichment analyse within the third latent variable using WebGestalt.

Supplementary Table 9. Gene set enrichment analysis: biological processes enriched in genes associated with cortical volume and cortical surface area in DLB

GO identifier	GO term	Gene set size	Number of leading edge IDs	ES	NES	*P*-value (FDR)
**Cortical Volume**						
**Negatively weighted genes (associated with lower cortical volume)**						
GO:0099003	vesicle-mediated transport in synapse	205	77	-0.47	-2.52	< 0.0001
GO:0010257	NADH dehydrogenase complex assembly	54	39	-0.56	-2.39	< 0.0001
GO:0099565	chemical synaptic transmission, postsynaptic	94	39	-0.49	-2.32	< 0.0001
GO:0022900	electron transport chain	146	87	-0.45	-2.27	< 0.0001
GO:0099177	regulation of trans-synaptic signaling	425	159	-0.38	-2.25	0.0002
GO:0007005	mitochondrion organization	439	197	-0.37	-2.15	0.002
GO:0070585	protein localization to mitochondrion	122	56	-0.43	-2.15	0.002
GO:0099072	regulation of postsynaptic membrane neurotransmitter receptor levels	82	37	-0.45	-2.10	0.002
GO:0070050	neuron cellular homeostasis	55	24	-0.49	-2.08	0.003
GO:0071867	response to monoamine	61	24	-0.47	-2.06	0.003
GO:0002209	behavioral defense response	41	17	-0.52	-2.05	0.004
GO:0098664	G protein-coupled serotonin receptor signaling pathway	19	9	-0.62	-2.04	0.004
GO:1901293	nucleoside phosphate biosynthetic process	240	99	-0.37	-2.03	0.004
GO:0001963	synaptic transmission, dopaminergic	23	11	-0.59	-2.02	0.004
GO:0015980	energy derivation by oxidation of organic compounds	280	115	-0.36	-2.00	0.005
GO:0035249	synaptic transmission, glutamatergic	97	38	-0.41	-1.98	0.006
GO:0072522	purine-containing compound biosynthetic process	216	89	-0.37	1.97	0.006
GO:1902600	proton transmembrane transport	114	56	-0.40	-1.96	0.006
GO:0009141	nucleoside triphosphate metabolic process	223	101	-0.36	-1.96	0.006
GO:0048499	synaptic vesicle membrane organization	27	10	-0.53	-1.94	0.006
GO:0019693	ribose phosphate metabolic process	396	158	-0.32	-1.88	0.010
GO:0050808	synapse organization	440	163	-0.31	-1.86	0.011
GO:0042391	regulation of membrane potential	364	138	-0.32	-1.83	0.014

GO:0043161	proteasome-mediated ubiquitin-dependent protein catabolic process	412	153	-0.30	-1.79	0.018
GO:0072594	establishment of protein localization to organelle	400	140	-0.30	-1.75	0.021
GO:001605	vesicle organization	323	132	-0.30	-1.71	0.028
**Positively weighted genes (associated with higher cortical volume)**						
GO:0070828 †	heterochromatin organization	88	47	0.050	2.36	< 0.0001
GO:0070269 †	pyroptosis	25	16	0.57	2.01	0.025
GO:0035329	hippo signaling	35	15	0.49	1.96	0.031
GO:0072337 †	modified amino acid transport	35	14	0.50	1.94	0.029
GO:0007606	sensory perception of chemical stimulus	107	55	0.38	1.89	0.038
**Cortical surface area**						
Positively weighted genes (associated with increased surface area)						
GO:0022900	electron transport chain	146	87	0.46	2.28	0.001
GO:0042060	wound healing	310	126	0.38	2.11	0.008
GO:1901657	glycosyl compound metabolic process	59	20	0.48	2.08	0.006
GO:0019882	antigen processing and presentation	92	34	0.45	2.06	0.006
GO:0002396	MHC protein complex assembly	18	11	0.65	2.03	0.007
GO:0036314	response to sterol	26	12	0.56	1.96	0.016
GO:0002347	response to tumour cell	35	13	0.52	1.95	0.015
GO:0050866	negative regulation of cell activation	139	67	0.40	1.95	0.014
GO:0044282	small molecule catabolic process	292	120	0.36	1.94	0.014
GO:0048010	vascular endothelial growth factor receptor signaling pathway	50	21	0.47	1.92	0.018
GO:0045785	positive regulation of cell adhesion	362	153	0.34	1.90	0.020
GO:0042063	gliogenesis	292	110	0.35	1.89	0.023
GO:0034109	homotypic cell-cell adhesion	67	32	0.44	1.88	0.024
GO:0050817	coagulation	161	62	0.37	1.83	0.037
GO:0032637	interleukin-8 production	55	35	0.44	1.83	0.037
GO:1901342	regulation of vasculature development	223	75	0.35	1.82	0.036
GO:0090594	inflammatory response to wounding	18	14	0.57	1.81	0.038
GO:0007229	integrin-mediated signaling pathway	91	47	0.39	1.80	0.040
GO:0050900	leukocyte migration	264	104	0.34	1.80	0.039
GO:0002181	cytoplasmic translation	148	63	0.36	1.80	0.037
GO:0072593	reactive oxygen species metabolic process	166	69	0.35	1.77	0.048
GO:0060840	artery development	85	38	0.39	1.76	0.049
GO:0006882	intracellular zinc ion homeostasis	28	13	0.49	1.76	0.049

GO:0035924	cellular response to vascular endothelial growth factor stimulus	53	29	0.42	1.75	0.048
GO:0050673	epithelial cell proliferation	319	92	0.32	1.75	0.049
GO:0010463	mesenchymal cell proliferation	34	14	0.46	1.74	0.049

List of genes significantly enriched in biological processes within the first latent variable for cortical volume and surface area based on gene enrichment analyse within the third latent variable using WebGestalt. † Result not found with Panther.

DLB, dementia with Lewy bodies; GO, gene ontology; FDR, false-discovery rate; ES, enrichment score; NES, normalized enrichment score.

Supplementary Table 10. Over-representation results of unique and shared genes in pathway enrichment of cortical atrophy in DLB

GO identifier	GO term	Gene set size	Expected value	Overlap	ER	P_{FDR}-value
**Common pathway genes**						
GO:0010257	NADH dehydrogenase complex assembly	54	0.13	22	166.53	**1.43E-45**
GO:0022900	electron transport chain	146	0.36	25	69.99	**2.72E-43**
GO:0072522	purine-containing compound biosynthetic process	216	0.53	27	51.09	**5.42E-44**
GO:0009141	nucleoside triphosphate metabolic process	223	0.55	27	49.49	**7.66E-44**
GO:1901293	nucleoside phosphate biosynthetic process	240	0.59	27	45.98	**4.18E-43**
GO:0015980	energy derivation by oxidation of organic compounds	280	0.69	28	40.88	**5.42E-44**
GO:0019693	ribose phosphate metabolic process	396	0.97	27	27.87	**4.86E-37**
GO:0007005	mitochondrion organization	439	1.07	22	20.48	**1.33E-24**
**Unique AD-pathway genes**						
GO:0050435	amyloid-beta metabolic process	48	0.13	6	44.71	**1.65E-06**
GO:0042982	amyloid precursor protein metabolic process	71	0.20	6	30.22	**7.15E-06**
GO:0007422	peripheral nervous system development	75	0.21	6	28.61	**7.15E-06**
GO:2000027	regulation of animal organ morphogenesis	65	0.18	5	27.51	**7.69E-05**
GO:0042063	gliogenesis	292	0.82	10	12.25	**1.65E-06**
GO:0051402	neuron apoptotic process	234	0.65	8	12.23	**2.24E-05**
GO:1903829	positive regulation of protein localization	415	1.16	11	9.48	**2.27E-06**
GO:0198738	cell-cell signaling by wnt	379	1.06	10	9.44	**7.15E-06**
GO:0002009	morphogenesis of an epithelium	388	1.08	9	8.30	**7.69E-05**
GO:0007409	axonogenesis	397	1.11	9	8.11	**7.69E-05**
**Unique PD-pathway genes**						

GO:0001963	synaptic transmission, dopaminergic	23	0.04	5	124.40	**2.62E-07**
GO:0098780	response to mitochondrial depolarisation	20	0.03	3	85.84	**4.76E-04**
GO:0015844	monoamine transport	70	0.12	5	40.88	**2.99E-05**
GO:0018958	phenol-containing compound metabolic process	81	0.14	4	28.26	**7.25E-04**
GO:0030534	adult behavior	121	0.21	5	23.65	**2.07E-04**
GO:0070585	protein localization to mitochondrion	122	0.21	5	23.45	**2.07E-04**
GO:0018209	peptidyl-serine modification	175	0.31	6	19.62	**6.39E-05**
GO:0051235	maintenance of location	265	0.46	7	15.12	**4.14E-05**
GO:0097193	intrinsic apoptotic signaling pathway	264	0.46	6	13.01	**4.41E-04**
GO:0042391	regulation of membrane potential	364	0.64	8	12.58	**2.99E-05**
**Shared AD- and PD-pathway genes**						
GO:0006882	intracellular zinc ion homeostasis	28	0.01	2	136.25	**0.0282**
GO:1990000	amyloid fibril formation	34	0.02	2	112.21	**0.0282**
GO:0002269	leukocyte activation involved in inflammatory response	38	0.20	2	100.39	**0.0282**
GO:0019755	one-carbon compound transport	39	0.02	2	97.82	**0.0282**
GO:0000041	transition metal ion transport	80	0.04	3	71.53	**0.0054**
GO:0150076	neuroinflammatory response	57	0.03	2	66.93	**0.0303**
GO:0051347	positive regulation of transferase activity	273	0.14	3	20.96	**0.0303**
GO:0051345	positive regulation of hydrolase activity	287	0.15	3	19.94	**0.0303**
GO:0010563	negative regulation of phosphorus metabolic process	292	0.15	3	19.60	**0.0303**
GO:0010038	response to metal ion	299	0.16	3	19.14	**0.0303**

Bold values represent significantly enriched biological process terms for every pathway enrichment analysis. Only the top 10 genes are shown when significant. Genes are ordered per analysis by decreasing enrichment scores.

AD, Alzheimer's disease; DLB, dementia with Lewy bodies; ER, enrichment ratio; FDR, false discovery rate; GO, Gene Ontology; KEGG, Kyoto Encyclopedia of Genes and Genomes; PD = Parkinson's disease.

Supplementary Table 11. Over-representation results of biological processes enriched in the genes not part of KEGG AD and PD pathways

GO identifier	GO term	Gene set size	Expected value	Overlap	ER	P_{FDR}-value
GO:0007214	GABA signaling pathway	25	0.19	5	26.62	**1.05E-04**
GO:0070050	neuron cellular homeostasis	55	0.41	11	26.62	**5.70E-11**
GO:1902600	proton transmembrane transport	114	0.86	17	19.85	**5.16E-15**
GO:0006885	regulation of pH	81	0.61	10	16.3	**8.57E-08**
GO:0099003	vesicle-mediated transport in synapse	205	1.54	20	12.98	**1.24E-14**
GO:0006820	monoatomic anion transport	124	0.93	8	8.59	**3.32E-04**
GO:0006898	receptor-mediated endocytosis	206	1.55	11	7.11	**4.91E-05**
GO:0021700	developmental maturation	255	1.92	12	6.26	**4.91E-05**
GO:0016050	vesicle organization	323	2.43	15	6.18	**2.48E-06**
GO:0042391	regulation of membrane potential	364	2.74	13	4.75	**2.73E-04**

Bold values represent significantly enriched biological process terms. Only the top 10 genes are shown when significant. Genes are ordered by decreasing enrichment scores.

AD, Alzheimer's disease; ER, enrichment ratio; FDR, false discovery rate; GO, Gene Ontology; KEGG, Kyoto Encyclopedia of Genes and Genomes; PD, Parkinson's disease.

Supplementary Table 12. Comparisons between enrichment in brain vs. body tissues of genes enriched in DLB-related atrophy independent from AD and PD pathways.

Gene ID	Gene name	Mean brain TPM	Mean body TPM	log_2 fold change brain vs. body	P-value	FDR-adjusted q-values
ENSG00000104888.10	*SLC17A7*	246.57	2.21	6.27	0.00629149	**0.01415585**
ENSG00000213760.11	*ATP6V1G2*	286.00	2.92	6.19	0.00000001	**0.00000018**
ENSG00000162728.5	*KCNJ9*	63.81	0.17	5.79	0.00000001	**0.00000018**
ENSG00000162188.6	*GNG3*	108.31	1.28	5.58	0.00000001	**0.00000018**
ENSG00000126583.12	*PRKCG*	40.03	0.47	4.80	0.00000002	**0.00000018**
ENSG00000079841.19	*RIMS1*	21.22	0.40	3.99	0.00000002	**0.00000018**
ENSG00000163285.8	*GABRG1*	14.90	0.10	3.85	0.00000001	**0.00000018**
ENSG00000155511.18	*GRIA1*	29.72	1.19	3.81	0.00000002	**0.00000018**
ENSG00000152578.13	*GRIA4*	15.93	0.54	3.46	0.00000002	**0.00000018**
ENSG00000148408.14	*CACNA1B*	15.79	0.76	3.25	0.00000007	**0.00000036**
ENSG00000125675.20	*GRIA3*	21.63	1.75	3.04	0.00000004	**0.00000026**
ENSG00000132535.22	*DLG4*	77.70	9.00	2.98	0.00000014	**0.00000065**
ENSG00000011677.13	*GABRA3*	7.29	0.08	2.94	0.00000000	**0.00000018**
ENSG00000152822.14	*GRM1*	7.02	0.07	2.91	0.00000002	**0.00000018**
ENSG00000171130.19	*ATP6V0E2*	88.34	11.41	2.85	0.00000002	**0.00000018**
ENSG00000078295.17	*ADCY2*	20.26	2.55	2.58	0.00000004	**0.00000026**
ENSG00000154654.15	*NCAM2*	11.23	1.14	2.51	0.00000004	**0.00000026**
ENSG00000166206.15	*GABRB3*	10.29	1.04	2.47	0.00000012	**0.00000058**
ENSG00000106089.12	*STX1A*	67.45	12.69	2.32	0.00007538	**0.00021200**
ENSG00000168243.11	*GNG4*	18.88	3.73	2.07	0.00000067	**0.00000273**
ENSG00000107902.14	*LHPP*	54.67	12.72	2.02	0.00000005	**0.00000029**
ENSG00000171867.18	*PRNP*	277.61	103.23	1.42	0.00000084	**0.00000330**
ENSG00000163288.14	*GABRB1*	1.85	0.10	1.38	0.00000002	**0.00000018**
ENSG00000033627.17	*ATP6V0A1*	101.03	38.38	1.37	0.00000010	**0.00000052**
ENSG00000185386.15	*MAPK11*	50.15	19.17	1.34	0.00008542	**0.00022612**
ENSG00000069966.19	*GNB5*	13.57	5.81	1.10	0.00077279	**0.00187976**
ENSG00000114573.10	*ATP6V1A*	51.76	24.45	1.05	0.00008025	**0.00021887**
ENSG00000047249.18	*ATP6V1H*	53.06	27.10	0.94	0.00000780	**0.00002599**
ENSG00000204843.13	*DCTN1*	122.86	66.79	0.87	0.00006645	**0.00019292**
ENSG00000185883.12	*ATP6V0C*	388.13	220.40	0.81	0.00016720	**0.00041800**
ENSG00000176697.20	*BDNF*	1.43	0.42	0.77	0.01049381	**0.02303519**

ENSG00000100554.12	*ATP6V1D*	43.57	25.49	0.75	0.00000156	**0.00000560**
ENSG00000128524.5	*ATP6V1F*	230.08	141.10	0.70	0.00000353	**0.00001223**
ENSG00000105258.9	*POLR2I*	99.43	61.91	0.67	0.00000020	**0.00000086**
ENSG00000007174.18	*DNAH9*	0.96	0.25	0.65	0.00000098	**0.00000369**
ENSG00000131100.13	*ATP6V1E1*	207.39	131.89	0.65	0.00009672	**0.00024870**
ENSG00000078369.18	*GNB1*	248.32	164.20	0.59	0.00144673	**0.00342647**
ENSG00000006125.18	*AP2B1*	75.82	55.28	0.45	0.00365359	**0.00843136**
ENSG00000188130.14	*MAPK12*	12.61	8.97	0.45	0.01902725	**0.03982448**
ENSG00000183914.15	*DNAH2*	0.82	0.37	0.41	0.00001791	**0.00005558**
ENSG00000179520.11	*SLC17A8*	0.46	0.16	0.33	0.00001477	**0.00004746**
ENSG00000100246.13	*DNAL4*	17.87	15.88	0.16	0.01902725	**0.03982448**

Bold values represent significant differences in the proportion of brain/CNS tissues (N=13) overexpressing the gene compared to non-brain tissues (N=55).

AD, Alzheimer's disease; DLB, dementia with Lewy bodies; ER, enrichment ratio; FDR, false discovery rate; PD, Parkinson's disease; TPM, transcripts per million.

Supplementary Table 13. Over-representation results of biological processes enriched in the genes preferentially expressed in brain tissues based on GTEx

GO identifier	GO term	Gene set size	Expected value	Overlap	ER	P_{FDR}-value
GO:0099177	regulation of trans-synaptic signaling	11	5.37	11	2.05	**0.0089**
GO:0042391	regulation of membrane potential	13	6.35	11	1.73	0.13
GO:0035249	synaptic transmission, glutamatergic	6	2.93	6	2.05	0.14
GO:0050808	synapse organization	6	2.93	6	2.05	0.14
GO:0006836	neurotransmitter transport	5	2.44	5	2.05	0.14
GO:0007215	glutamate receptor signaling pathway	5	2.44	5	2.05	0.14
GO:0050803	regulation of synapse structure or activity	5	2.44	5	2.05	0.14
GO:0098739	import across plasma membrane	5	2.44	5	2.05	0.14
GO:0006820	monoatomic anion transport	8	3.91	7	1.79	0.14
GO:0007033	vacuole organization	7	3.42	6	1.76	0.22

Bold values represent significantly enriched biological process terms. Only the top 10 genes are shown.

DLB, dementia with Lewy bodies; ER, enrichment ratio; FDR, false discovery rate; GO, Gene Ontology; GTEx, Genotype-Tissue Expression.

Supplementary Table 14. Results of spatial mapping between neurochemical maps and cortical thickness in DLB

Transmitter	Spearman correlation coefficient	P-value	P_{FDR}-corrected value	P-value test against random null model	P-value test against spatial null model
**$5\text{-}HT_{1A}$**	-0.35	0.0034	**0.014**	**0.0001**	**0.0169**
**$5\text{-}HT_{1B}$**	0.43	0.00026	**0.002**	**0.0003**	**0.0041**
**$5\text{-}HT_{2A}$**	-0.01	0.91	0.96	-	-
**$5\text{-}HT_{4}$**	-0.48	0.00004	**0.0004**	**0.0001**	**0.0002**
**$5\text{-}HT_{6}$**	0.01	0.89	0.96	-	-
**5-HTT**	0.20	0.095	0.24	-	-
**α4β2**	0.21	0.081	0.23	-	-
**CB_1**	-0.17	0.161	0.36	-	-
**D_1**	0.15	0.22	0.44	-	-
**D_2**	-0.52	0.000007	**<0.0001**	**0.0001**	**0.0003**
**DAT**	0.09	0.456	0.70	-	-
**$GABA_A$**	0.36	0.0025	**0.013**	**0.0014**	**0.012**
**H_3**	0.23	0.054	0.18	-	-
**M_1**	-0.12	0.34	0.62	-	-
**mGluR5**	-0.01	0.97	0.97	-	-
**μ**	0.02	0.86	0.96	-	-
**NET**	0.08	0.50	0.72	-	-
**NMDA**	0.04	0.73	0.96	-	-
**VAChT**	0.109	0.37	0.62	-	-
**SV2A**	-0.01	0.91	0.96	-	-

Empirical Spearman correlation coefficients for the associations between receptors, transporters, and receptor binding sites of different neurotransmitter systems and cortical thickness W-scores in DLB. P-values were corrected for FDR correction. Significant correlations were further tested against distributions of correlations from sets of random and spatial null models.

DLB, dementia with Lewy bodies; FDR, false discovery rate.

References

1. Chertkow H, Borrie M, Whitehead V, et al. The Comprehensive Assessment of Neurodegeneration and Dementia: Canadian Cohort Study. *Can J Neurol Sci*. Sep 2019;46(5):499-511.
2. Duchesne S, Chouinard I, Potvin O, et al. The Canadian Dementia Imaging Protocol: Harmonizing National Cohorts. *J Magn Reson Imaging*. Feb 2019;49(2):456-465.
3. Mohaddes Z, Das S, Abou-Haidar R, et al. National Neuroinformatics Framework for Canadian Consortium on Neurodegeneration in Aging (CCNA). *Front Neuroinform*. 2018;12:85.
4. Potvin O, Chouinard I, Dieumegarde L, et al. The Canadian Dementia Imaging Protocol: Harmonization validity for morphometry measurements. *Neuroimage Clin*. 2019;24:101943.
5. Donaghy PC, Firbank MJ, Thomas AJ, et al. Clinical and imaging correlates of amyloid deposition in dementia with Lewy bodies. *Mov Disord*. Jul 2018;33(7):1130-1138.
6. Firbank M, Kobeleva X, Cherry G, et al. Neural correlates of attention-executive dysfunction in lewy body dementia and Alzheimer's disease. *Hum Brain Mapp*. Mar 2016;37(3):1254-70.
7. Taylor JP, Firbank MJ, He J, et al. Visual cortex in dementia with Lewy bodies: magnetic resonance imaging study. *Br J Psychiatry*. Jun 2012;200(6):491-8.
8. Nasreddine ZS, Phillips NA, Bedirian V, et al. The Montreal Cognitive Assessment, MoCA: a brief screening tool for mild cognitive impairment. *J Am Geriatr Soc*. Apr 2005;53(4):695-9.
9. Folstein MF, Folstein SE, McHugh PR. "Mini-mental state". A practical method for grading the cognitive state of patients for the clinician. *J Psychiatr Res*. Nov 1975;12(3):189-98.
10. Ferman TJ, Smith GE, Boeve BF, et al. DLB fluctuations: specific features that reliably differentiate DLB from AD and normal aging. *Neurology*. Jan 27 2004;62(2):181-7.
11. Lee DR, McKeith I, Mosimann U, et al. The dementia cognitive fluctuation scale, a new psychometric test for clinicians to identify cognitive fluctuations in people with dementia. *Am J Geriatr Psychiatry*. Sep 2014;22(9):926-35.
12. Cummings JL, Mega M, Gray K, Rosenberg-Thompson S, Carusi DA, Gornbein J. The Neuropsychiatric Inventory: comprehensive assessment of psychopathology in dementia. *Neurology*. Dec 1994;44(12):2308-14.
13. Donaghy PC, Barnett N, Olsen K, et al. Symptoms associated with Lewy body disease in mild cognitive impairment. *Int J Geriatr Psychiatry*. Nov 2017;32(11):1163-1171.
14. Mosimann UP, Collerton D, Dudley R, et al. A semi-structured interview to assess visual hallucinations in older people. *Int J Geriatr Psychiatry*. Jul 2008;23(7):712-8.
15. Goetz CG, Tilley BC, Shaftman SR, et al. Movement Disorder Society-sponsored revision of the Unified Parkinson's Disease Rating Scale (MDS-UPDRS): scale presentation and clinimetric testing results. *Mov Disord*. Nov 15 2008;23(15):2129-70.
16. Movement Disorder Society Task Force on Rating Scales for Parkinson's D. The Unified Parkinson's Disease Rating Scale (UPDRS): status and recommendations. *Mov Disord*. Jul 2003;18(7):738-50.
17. Norgaard M, Beliveau V, Ganz M, et al. A high-resolution in vivo atlas of the human brain's benzodiazepine binding site of GABA(A) receptors. *Neuroimage*. May 15 2021;232:117878.

18. Hansen JY, Shafiei G, Markello RD, et al. Mapping neurotransmitter systems to the structural and functional organization of the human neocortex. *Nat Neurosci*. Nov 2022;25(11):1569-1581.
19. DuBois JM, Rousset OG, Rowley J, et al. Characterization of age/sex and the regional distribution of mGluR5 availability in the healthy human brain measured by high-resolution [(11)C]ABP688 PET. *Eur J Nucl Med Mol Imaging*. Jan 2016;43(1):152-162.
20. Smart K, Cox SML, Scala SG, et al. Sex differences in [(11)C]ABP688 binding: a positron emission tomography study of mGlu5 receptors. *Eur J Nucl Med Mol Imaging*. May 2019;46(5):1179-1183.
21. Hillmer AT, Esterlis I, Gallezot JD, et al. Imaging of cerebral alpha4beta2* nicotinic acetylcholine receptors with (-)-[(18)F]Flubatine PET: Implementation of bolus plus constant infusion and sensitivity to acetylcholine in human brain. *Neuroimage*. Nov 1 2016;141:71-80.
22. Naganawa M, Nabulsi N, Henry S, et al. First-in-Human Assessment of (11)C-LSN3172176, an M1 Muscarinic Acetylcholine Receptor PET Radiotracer. *J Nucl Med*. Apr 2021;62(4):553-560.
23. Aghourian M, Legault-Denis C, Soucy JP, et al. Quantification of brain cholinergic denervation in Alzheimer's disease using PET imaging with [(18)F]-FEOBV. *Mol Psychiatry*. Nov 2017;22(11):1531-1538.
24. Bedard MA, Aghourian M, Legault-Denis C, et al. Brain cholinergic alterations in idiopathic REM sleep behaviour disorder: a PET imaging study with (18)F-FEOBV. *Sleep Med*. Jun 2019;58:35-41.
25. Kaller S, Rullmann M, Patt M, et al. Test-retest measurements of dopamine D(1)-type receptors using simultaneous PET/MRI imaging. *Eur J Nucl Med Mol Imaging*. Jun 2017;44(6):1025-1032.
26. Jaworska N, Cox SML, Tippler M, et al. Extra-striatal D(2/3) receptor availability in youth at risk for addiction. *Neuropsychopharmacology*. Aug 2020;45(9):1498-1505.
27. Smith CT, Crawford JL, Dang LC, et al. Partial-volume correction increases estimated dopamine D2-like receptor binding potential and reduces adult age differences. *J Cereb Blood Flow Metab*. May 2019;39(5):822-833.
28. Sandiego CM, Gallezot JD, Lim K, et al. Reference region modeling approaches for amphetamine challenge studies with [11C]FLB 457 and PET. *J Cereb Blood Flow Metab*. Mar 31 2015;35(4):623-9.
29. Alakurtti K, Johansson JJ, Joutsa J, et al. Long-term test-retest reliability of striatal and extrastriatal dopamine D2/3 receptor binding: study with [(11)C]raclopride and high-resolution PET. *J Cereb Blood Flow Metab*. Jul 2015;35(7):1199-205.
30. Sasaki T, Ito H, Kimura Y, et al. Quantification of dopamine transporter in human brain using PET with 18F-FE-PE2I. *J Nucl Med*. Jul 2012;53(7):1065-73.
31. Marek K, Chowdhury S, Siderowf A, et al. The Parkinson's progression markers initiative (PPMI) - establishing a PD biomarker cohort. *Ann Clin Transl Neurol*. Dec 2018;5(12):1460-1477.
32. Hesse S, Becker GA, Rullmann M, et al. Central noradrenaline transporter availability in highly obese, non-depressed individuals. *Eur J Nucl Med Mol Imaging*. Jun 2017;44(6):1056-1064.
33. Ding YS, Singhal T, Planeta-Wilson B, et al. PET imaging of the effects of age and cocaine on the norepinephrine transporter in the human brain using (S,S)-[(11)C]O-methylreboxetine and HRRT. *Synapse*. Jan 2010;64(1):30-8.

34. Beliveau V, Ganz M, Feng L, et al. A High-Resolution In Vivo Atlas of the Human Brain's Serotonin System. *J Neurosci*. Jan 4 2017;37(1):120-128.
35. Savli M, Bauer A, Mitterhauser M, et al. Normative database of the serotonergic system in healthy subjects using multi-tracer PET. *Neuroimage*. Oct 15 2012;63(1):447-59.
36. Gallezot JD, Nabulsi N, Neumeister A, et al. Kinetic modeling of the serotonin 5-HT(1B) receptor radioligand [(11)C]P943 in humans. *J Cereb Blood Flow Metab*. Jan 2010;30(1):196-210.
37. Talbot PS, Slifstein M, Hwang DR, et al. Extended characterisation of the serotonin 2A (5-HT2A) receptor-selective PET radiotracer 11C-MDL100907 in humans: quantitative analysis, test-retest reproducibility, and vulnerability to endogenous 5-HT tone. *Neuroimage*. Jan 2 2012;59(1):271-85.
38. Radhakrishnan R, Nabulsi N, Gaiser E, et al. Age-Related Change in 5-HT(6) Receptor Availability in Healthy Male Volunteers Measured with (11)C-GSK215083 PET. *J Nucl Med*. Sep 2018;59(9):1445-1450.
39. Laurikainen H, Tuominen L, Tikka M, et al. Sex difference in brain CB1 receptor availability in man. *Neuroimage*. Jan 1 2019;184:834-842.
40. Normandin MD, Zheng MQ, Lin KS, et al. Imaging the cannabinoid CB1 receptor in humans with [11C]OMAR: assessment of kinetic analysis methods, test-retest reproducibility, and gender differences. *J Cereb Blood Flow Metab*. Aug 2015;35(8):1313-22.
41. Gallezot JD, Planeta B, Nabulsi N, et al. Determination of receptor occupancy in the presence of mass dose: [(11)C]GSK189254 PET imaging of histamine H(3) receptor occupancy by PF-03654746. *J Cereb Blood Flow Metab*. Mar 2017;37(3):1095-1107.
42. Mecca AP, Chen MK, O'Dell RS, et al. In vivo measurement of widespread synaptic loss in Alzheimer's disease with SV2A PET. *Alzheimers Dement*. Jul 2020;16(7):974-982.
43. Holmes SE, Scheinost D, Finnema SJ, et al. Lower synaptic density is associated with depression severity and network alterations. *Nat Commun*. Apr 4 2019;10(1):1529.
44. Finnema SJ, Rossano S, Naganawa M, et al. A single-center, open-label positron emission tomography study to evaluate brivaracetam and levetiracetam synaptic vesicle glycoprotein 2A binding in healthy volunteers. *Epilepsia*. May 2019;60(5):958-967.
45. Chen MK, Mecca AP, Naganawa M, et al. Assessing Synaptic Density in Alzheimer Disease With Synaptic Vesicle Glycoprotein 2A Positron Emission Tomographic Imaging. *JAMA Neurol*. Oct 1 2018;75(10):1215-1224.
