# Supplementary material for: Selective molecular and network architecture features underlie brain cortical atrophy in dementia with Lewy bodies
Source: J Biomed Sci. 2026 Jun 10;33:61. doi: 10.1186/s12929-026-01267-6 (PMC13251274; doi:10.1186/s12929-026-01267-6)
Supplement: Supplementary file 3 — Supplementary material 3. [file 12929_2026_1267_MOESM3_ESM.pdf]

# Supplementary methods

## MRI acquisition and processing

T1-weighted MRI scans were acquired at each centre. Participants in the Newcastle cohort were scanned using a 3T Philips Achieva system using an 8-channel head coil. Two MPRAGE protocols were used: 1) TR = 8.3 ms, TE = 4.6 ms, flip angle = 8°, inversion time = 1250 ms, field of view (FOV) = 216 x 240 mm, voxel size = 1 x 1 x 1 mm; or 2) TR = 9.6 ms, TE = 4.6 ms, flip angle = 8°, inversion time = 1250 ms, FOV = 240 x 240 mm, voxel size = 0.96 x 0.96 x 0.96 mm. Participants from the CCNA cohort were scanned using the standardized Canadian Dementia Imaging Protocol,[1, 2] designed to harmonize acquisitions across sites. Full acquisition parameters for CCNA centers have been described previously.[1]

All T1-weighted scans were processed using FreeSurfer (v7.1.1) to generate vertex-wise maps of cortical thickness, surface area, and cortical volume, following procedures from previous studies.[3, 4] Processing included skull stripping,[5] Talairach transformation, intensity normalization,[6] and grey/white matter boundary tessellation. Automated topology correction[7, 8] and surface deformation were performed to refine the placement of grey-white and pial surfaces using intensity gradients.[9-11] Subsequent steps included surface inflation,[12] spherical registration based on cortical folding patterns,[13] anatomical parcellation,[14, 15] and creation of surface-based datasets. Cortical thickness was defined as the shortest distance between the white and pial surfaces at each vertex.[10] Thickness estimates have been validated against histological measures[16] and manual tracings.[17, 18] All reconstructions were visually inspected and manually corrected slice by slice by trained raters (A.De., S.J., S.R.). Maps with major segmentation errors (rating > 2 on a 4-point scale) were excluded.[19, 20] Of the 178 available scans (89 DLB, 89 age- and sex-matched controls), 9 (5%) were excluded due to failed FreeSurfer processing or quality control (6 DLB, 3 controls).

## References


1. Duchesne S, Chouinard I, Potvin O, et al. The Canadian Dementia Imaging Protocol: Harmonizing National Cohorts. *J Magn Reson Imaging*. Feb 2019;49(2):456-465.

2. Potvin O, Chouinard I, Dieumegarde L, et al. The Canadian Dementia Imaging Protocol: Harmonization validity for morphometry measurements. *Neuroimage Clin*. 2019;24:101943.

3. Rahayel S, Tremblay C, Vo A, et al. Brain atrophy in prodromal synucleinopathy is shaped by structural connectivity and gene expression. *Brain*. Sep 14 2022;145(9):3162-3178.

4. Rahayel S, Tremblay C, Vo A, et al. Mitochondrial function-associated genes underlie cortical atrophy in prodromal synucleinopathies. *Brain*. Aug 1 2023;146(8):3301-3318.

5. Segonne F, Dale AM, Busa E, et al. A hybrid approach to the skull stripping problem in MRI. *Neuroimage*. Jul 2004;22(3):1060-75.

6. Sled JG, Zijdenbos AP, Evans AC. A nonparametric method for automatic correction of intensity nonuniformity in MRI data. *IEEE Trans Med Imaging*. Feb 1998;17(1):87-97.

7. Segonne F, Pacheco J, Fischl B. Geometrically accurate topology-correction of cortical surfaces using nonseparating loops. *IEEE Trans Med Imaging*. Apr 2007;26(4):518-29.

8. Fischl B, Liu A, Dale AM. Automated manifold surgery: constructing geometrically accurate and topologically correct models of the human cerebral cortex. *IEEE Trans Med Imaging*. Jan 2001;20(1):70-80.

9. Dale AM, Fischl B, Sereno MI. Cortical surface-based analysis. I. Segmentation and surface reconstruction. *Neuroimage*. Feb 1999;9(2):179-94.

10. Fischl B, Dale AM. Measuring the thickness of the human cerebral cortex from magnetic resonance images. *Proc Natl Acad Sci U S A*. Sep 26 2000;97(20):11050-5.

11. Dale AM, Sereno MI. Improved Localizadon of Cortical Activity by Combining EEG and MEG with MRI Cortical Surface Reconstruction: A Linear Approach. *J Cogn Neurosci*. Spring 1993;5(2):162-76.

12. Fischl B, Sereno MI, Dale AM. Cortical surface-based analysis. II: Inflation, flattening, and a surface-based coordinate system. *Neuroimage*. Feb 1999;9(2):195-207.

13. Fischl B, Sereno MI, Tootell RB, Dale AM. High-resolution intersubject averaging and a coordinate system for the cortical surface. *Hum Brain Mapp*. 1999;8(4):272-84.

14. Desikan RS, Segonne F, Fischl B, et al. An automated labeling system for subdividing the human cerebral cortex on MRI scans into gyral based regions of interest. *Neuroimage*. Jul 1 2006;31(3):968-80.

15. Fischl B, van der Kouwe A, Destrieux C, et al. Automatically parcellating the human cerebral cortex. *Cereb Cortex*. Jan 2004;14(1):11-22.

16. Rosas HD, Liu AK, Hersch S, et al. Regional and progressive thinning of the cortical ribbon in Huntington's disease. *Neurology*. Mar 12 2002;58(5):695-701.

17. Salat DH, Buckner RL, Snyder AZ, et al. Thinning of the cerebral cortex in aging. *Cereb Cortex*. Jul 2004;14(7):721-30.

18. Kuperberg GR, Broome MR, McGuire PK, et al. Regionally localized thinning of the cerebral cortex in schizophrenia. *Arch Gen Psychiatry*. Sep 2003;60(9):878-88.

19. Klapwijk ET, van de Kamp F, van der Meulen M, Peters S, Wierenga LM. Qoala-T: A supervised-learning tool for quality control of FreeSurfer segmented MRI data. *Neuroimage*. Apr 1 2019;189:116-129.

20. Monereo-Sanchez J, de Jong JJA, Drenthen GS, et al. Quality control strategies for brain MRI segmentation and parcellation: Practical approaches and recommendations - insights from the Maastricht study. *Neuroimage*. Aug 15 2021;237:118174.
